# Mixed-Mode I-II Fracture Process Zone Characteristic of the Four-Point Shearing Concrete Beam

**DOI:** 10.3390/ma13143203

**Published:** 2020-07-18

**Authors:** Guodong Li, Zhengyi Ren, Jiangjiang Yu

**Affiliations:** 1 Transportation Institute, Inner Mongolia University, Hohhot 010070, China; justice0224@163.com (Z.R.); yujj0904@163.com (J.Y.); 2 Inner Mongolia Engineering Research Center of Testing and Strengthening for Bridges, Hohhot 010070, China

**Keywords:** four-point shearing concrete beam, mixed-mode I-II FPZ, DIC, coarse aggregate

## Abstract

The size of the fracture process zone (FPZ) has significance for studying the fracture mechanism and fracture characteristics of concrete. This paper presents the method of assessing the FPZ of Mixed-Mode I-II for quasi-static four-point shearing concrete beams with pre-notched by Lagrangian strain profiles from digital image correlation (DIC). Additionally, it explores the influences of volume rates of the coarse aggregate of 0%, 28%, 48%, and 68%, and the specific surface areas of 0.12 m^2^/kg, 0.15 m^2^/kg, and 0.26 m^2^/kg on the size of the FPZ. It shows that the size of FPZ in four-point shearing concrete beam can be characterized by the displacement field and strain field using DIC. The size of FPZ conforms to linear positive correlation with the volume rate of coarse aggregate, and linear negative correlation with the specific surface area of coarse aggregate. It presents that the crack initiation of the four-point shearing beam with the pre notch is dominated by mode I load, and the propagation and fracture of Mixed-Mode I-II cracks are caused by the combined effect of Mode I and Mode II loading.

## 1. Introduction

The fracture of concrete and other quasi-brittle materials may exhibit a significant nonlinear region surrounding the tip of macroscopic cracks, similar to the plastic zone at the tip of cracks in metal materials. Therefore, unlike the ideal brittle material, the crack initiation does not represent the beginning of instability. There is a long stable subcritical stage of crack propagation in the evolution of crack. The micro-crack zone and the subcritical propagation zone at the crack tip are called fracture process zone (FPZ), which is often considered to be the material property [1].

The formation mechanism of FPZ, such as micro-cracking, crack deflection, crack bridging, crack-tip blunting, and crack-branching, is very complicated [2,3,4]. The primary cause is a mutual influence between micro-cracks by heterogeneous property of the mesoscopic structure of concrete, and it constitutes the network of micro-cracks within a certain zone. This has an effect of degradation and shielding to the propagation of microscopic cracks, including micro crack initiation, detours around the crack, bridging toughening of aggregate, friction between crack surface, passivation, and bifurcation of the cracks. FPZ is closely related to the propagation of cracks, and the primary cause of the nonlinear fracture process of concrete. The evaluation of FPZ becomes critical in deciding the applicability of linear fracture mechanics [5,6,7]. The size of FPZ has significance for studying the mechanism and characteristics of concrete fracture.

Many experimental techniques have been used for investigating FPZ in concretes, which include direct and indirect approaches. The direct approaches involve the scanning electron microscopy (SEM) [8,9], the X-ray diffraction method [10,11,12], the laser-speckle interferometry [13,14], photo-elastic coating method [15], moire interferometry [16], acoustic emission (AE) [17,18], and digital image correlation (DIC) [3,4,7]. For the indirect approach, characteristics of the FPZ are found through the parametric fitting of experimental results, such as load-deflection or load-crack mouth opening displacement, without directly identifying the local fracture processes in the specimen [19,20]. However, both of the approaches have limitations. After the peak load, the crack propagation is unstable under the influence of environmental noise, sample surface treatment, vibration, or illumination, and the FPZ is difficult to be observed. However, the DIC has many advantages when compared with other optical techniques, such as ordinary incoherent light being sufficient, specimen preparation being simple, and a vibration isolation table with complicated optics being unnecessary. With the advent of high-speed digital cameras in recent years, recording rates as high as several million frames per second at a relatively high spatial resolution have become possible. The resulting DIC technique is feasible for evaluating the evolution process of FPZ. So far, the DIC technique has been successfully used for investigating crack propagations in various conditions, and the definition of the FPZ can be realized by the stress field [21,22], displacement field [23,24,25], and displacement asymptote matching procedure [26,27,28,29,30] of the micro-crack zone. Therefore, using the DIC method to directly observe the crack propagation, study the mechanical characteristics of concrete FPZ, and clarify its evolution law has important scientific and engineering application value for the establishment of complete fracture theory and practical engineering fracture model, as well as the evaluation of crack stability in engineering.

In practical engineering, there are many modes for cracks to propagate, such as Mode I (Opening Mode), Mode II (Sliding Mode), and Mode III (Tearing Mode) and the combinations of above modes. In mass concrete structures, the improvement of structural design eliminates the potential risk of Mode I cracks effectively. Therefore, the Mixed-Mode I-II cracks are the most common and dangerous mode of fracture failure in concrete structures due to the asymmetry of structures, loads, or materials [31]. The failure of Mixed-Mode I-II cracks is a kind of brittle fracture and the FPZ is very difficult to be fully captured at the moment of fracture. Additionally, it is easy to add the size of macro cracks into FPZ after the crack expansion. Therefore, the Lagrangian strain profiles of DIC was used in this study in order to measure the FPZ of Mixed-Mode I-II cracks in concrete. Therefore, studying the evolution mechanism of concrete FPZ under different mix-proportions plays an important role in understanding and controlling the development of concrete cracks. It reveals the evolution of FPZ by exploring the developmental process of the displacement contour for the four-point shear experiments. Additionally, it explores the influences of volume rates of the coarse aggregate of 0%, 28%, 48%, and 68%, and specific surface areas of 0.12 m^2^/kg, 0.15 m^2^/kg, and 0.26 m^2^/kg on the FPZ, which provides the characterization of the influence of coarse aggregate on the fracture characteristics of the concrete at the mesoscale.

## 2. Experimental Program

### 2.1. Materials and Mixture Proportions

The materials used in this study include: P.O 42.5 ordinary Portland cement, I-type fly ash, coarse aggregate, and fine aggregate. Table 1, Table 2 and Table 3 show the chemical constituent and phase composition of the cement and fly ash [30]. It designs four kinds of mix proportions with the volume rates of coarse aggregate of 0%, 28%, 48%, and 68%, and three kinds of mix proportions with the specific surface area of coarse aggregate 0.12 m^2^/kg, 0.15 m^2^/kg, and 0.26 m^2^/kg. the volume rate of coarse aggregate is shown in Equation (1) and the specific surface area of coarse aggregate is shown in Equation (2). The water to binder rate is 0.34 and the slump value is 180 ± 20 mm. All of the specimens are cured for 28 days with temperature of 20 ± 2 °C and humidity above 95%. Table 4 shows the mix proportions of concrete and the compressive strength [31].

(1) $$V=\frac{m}{\rho }$$

where *m* is the mass of coarse aggregate and *ρ* is the natural accumulation density of coarse aggregate.

(2) $$S=\sum_{1}^{4}n_{i}S_{i}$$

where, *S_i_* is the approximately spherical surface area of coarse aggregate with single particle size *i*, *i* is the aperture size (4.75 mm, 9.5 mm, 19 mm, 26.5 mm, and 31.5 mm), *S_i_* is shown in Equation (3).

(3) $$S_{i}={\left(\frac{d_{i}+d_{i+1}}{4}\right)}^{2}4\pi$$

*n_i_* is the particle number of coarse aggregate with particle size *i* in unit volume, as shown in Equation (4).

(4) $$n_{i}=\frac{M_{i}}{m_{i}}$$

where *M_i_* is the mass of coarse aggregate with particle size *i* in unit volume and *m_i_* is the mass of coarse aggregate with particle size *i*.

(5) $$M_{i}=P\left(i\right)M;\;m_{i}=\rho \cdot \frac{4}{3}\pi {\left(\frac{d_{i}+d_{i+1}}{4}\right)}^{3}$$

where *M* is the total mass of coarse aggregate in unit volume and *ρ* is the density of coarse aggregate. *P*(*i*) is the percentage of accumulated sieve residues.

### 2.2. Digital Image Correlation (DIC)

**Figure 1:**
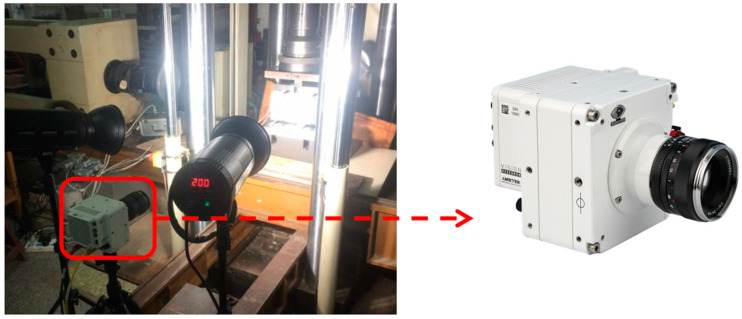
High-speed photography camera.

DIC is simply a particle tracking method that can be used to determine the displacements of particles (speckles) in a digital image using the high-speed camera (VEO 1310, Vision Research Company, Wayne, United States) shown in Figure 1. More specifically, DIC refers to a class of non-contacting optical approaches that analyze digital images to extract full-field displacement of the specimen surface. The numerical analysis of digital images is based on the matching of a small region called a subset [12]. A variety of numerical algorithms, including the Newton-Raphson method, have been developed through minimizing a correlation coefficient of intensity values in the subset between two digital images, to obtain full-field in-plane displacement [8,13]. During DIC processing, there are two digital images, reference and current, recorded with respect to the different loading conditions. The displacement and strain can be measured by identifying the discrete function of two digital gray fields. The reference image representing the body before distortion is a discrete function *f* (*X*,*Y*) and it is transformed into another discrete function *g* (*X*′,*Y*′) after distortion or displacement. The theoretical relation between the two discrete functions can be written, as follows:

(6) $$g(X^{\prime },Y^{\prime })-f(X+u(X,Y),Y+v(X,Y))=0$$

where, *u*(*X,Y*) and *v*(*X,Y*) denote the displacement field for a pattern.

Because of the randomness of the speckle pattern, the target pattern is found through the speckle intensity distribution and sample pattern, and the deformation measurement will be converted to correlation calculation. The correlation coefficient calculation formula as the criterion is shown in Equation (7).

(7) $$C=\frac{\sum f(X,Y)\cdot \sum g(X^{\prime },Y^{\prime })}{\sqrt{\sum f^{2}(X,Y)\cdot \sum g^{2}(X^{\prime },Y^{\prime })}}$$

where, *C* = 1 means completely relevant, while *C* = 0 means completely irrelevant. DIC gets the incremental displacement of the concrete four-point shear beam by comparing the reference and current images with different loads. The horizontal displacement *U* and the vertical displacement *V* in displacement field are both obtained through the calculation of the minimum correlation coefficient. The displacement field is given by Equation (8) and the Lagrangian strain field is obtained using Equations (9)–(11) [32,33,34].

(8) $$\left\{ \begin{array}{l} U_{i}=Lu_{i}\\ V_{i}=Lv_{i} \end{array}\right.$$

(9) $$e_{XX}=\frac{\partial u}{\partial X}+\frac{1}{2}\left[{\left(\frac{\partial u}{\partial X}\right)}^{2}+{\left(\frac{\partial v}{\partial X}\right)}^{2}\right]$$

(10) $$e_{YY}=\frac{\partial v}{\partial Y}+\frac{1}{2}\left[{\left(\frac{\partial u}{\partial Y}\right)}^{2}+{\left(\frac{\partial v}{\partial Y}\right)}^{2}\right]$$

(11) $$e_{XY}=\frac{1}{2}\left(\frac{\partial u}{\partial Y}+\frac{\partial v}{\partial X}\right)+\frac{1}{2}\left(\frac{\partial u}{\partial X}\frac{\partial u}{\partial Y}+\frac{\partial v}{\partial X}\frac{\partial V}{\partial Y}\right)$$

where,

*U_i_*—horizontal displacement,
*V_i_*—vertical displacement,
*u_i_*—horizontal displacement of pixels in an image,
*v_i_*—vertical displacement of pixels in an image,
*e_XX_*—horizontal strain in Lagrangian strain field,
*e_YY_*—vertical strain in Lagrange strain field,
*e_XY_*—shear strain in Lagrangian strain field, and
*L*—large coefficient, the value of this paper is 200 μm/pixel.

### 2.3. Experimental Setup

**Figure 2:**
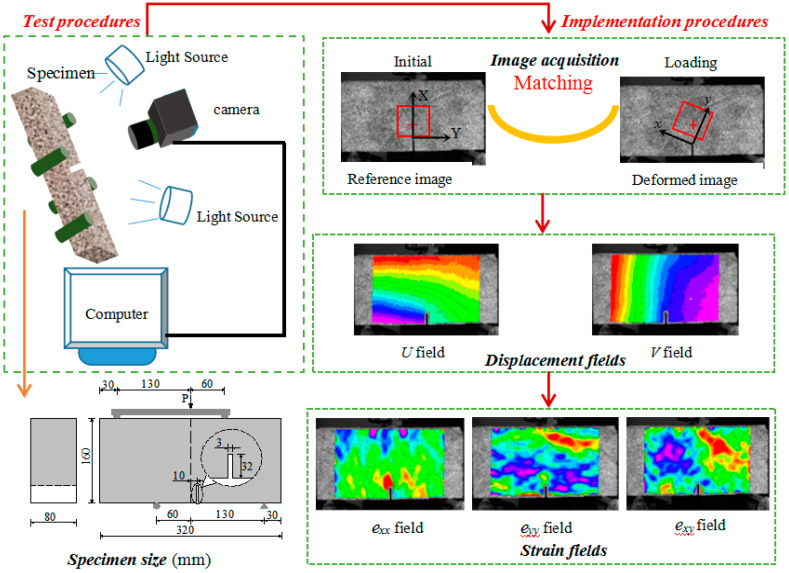
Test and implementation procedures.

The Mode I fracture in concrete can be realized by three-point bending test and wedge splitting experiment, the experimental method of Mode II fracture has been a difficult research topic up to now. There are both bending moment and shearing force at the top of the pre-notch in the four-point shearing beam, and it is easy to introduce two stress components of Mode I and Mode II into the specimen. Therefore, the four-point shearing test is a simple and feasible method for the theoretical and experimental study of Mixed-Mode I-II fracture. In this paper, a four-point shear beam with an off-center notch of 10 mm and a size of 320 mm × 160 mm × 80 mm was designed, as shown in Figure 2. The ratio of the notch height to the specimen height is 0.2. Crack initiation and propagation are controlled by the servo-hydraulic system with crack mouth opening displacement (CMOD) as the feedback signal at a rate of 0.005 mm/s. The CMOD was continuously measured using a displacement sensor and DIC. It measures and identifies the FPZ at the tip of the crack using the DIC. During the experiment process, the acquisition frequency of the speckle pattern was 300 frames/s before cracking, and 17,000 frames/s after crack initiation, and the different test phases were identified by the changing trend of Time-CMOD curves. Once the results of CMOD showed the trend of non-linear increase, the acquisition frequency of DIC was increased to 17,000 frames/s. Figure 2 shows the testing system. Three replicate beams were tested for each mix proportion.

## 3. Results and Discussions

### 3.1. Macroscopic Behavior and Response

**Figure 3:**
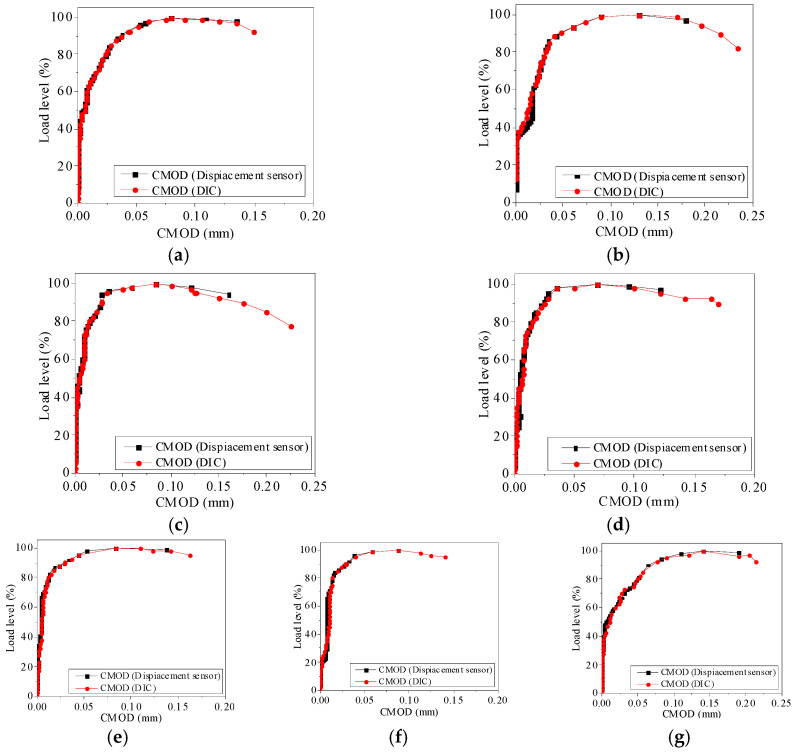
Verification results of DIC displacement accuracy. (**a**) S1; (**b**) S2; (**c**) S3; (**d**) S4; (**e**) S5; (**f**) S6; (**g**) S7.

**Figure 4:**
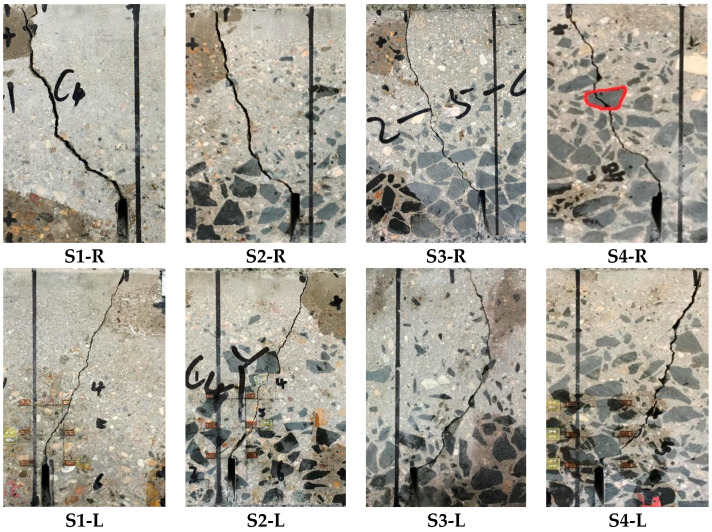
The fracture path of right and left planes of the specimen.

Figure 3 shows the Load-CMOD curves obtained by the displacement sensor and the DIC system. It can be seen that the compliance of the F-CMOD curve that is measured by DIC and displacement sensor is very high under different mix proportions. The F-CMOD curves of concrete mixed-mode I-II fracture are similar under different proportions, and the cracking load level of concrete specimens under seven proportions is 20–50%. After cracking, the slope of F-CMOD curve decreases gradually. When it approaches the peak load, the slope of the F-CMOD curve becomes 0. When it exceeds the peak load, the specimen enters the unloading stage, the load decreases, and the opening displacement continues to increase until the failure occurs. Because the two methods respectively tested two different planes of the specimen, and the fracture paths in two different planes of the specimen are quite different as shown in Figure 4. It can be seen from the figure that the fracture trend of the same sample is similar under the action of mixed-mode I-II stress, and the specific fracture path is different. Because of the randomness of aggregate distribution, the crack will preferentially expand from ITZ with weak strength. When the strength of individual aggregate particles is low, the crack will continue to expand through the aggregate. The accuracy of F-CMOD curve that is measured by DIC method is more than 95%.

### 3.2. The Size of the FPZ

**Figure 5:**
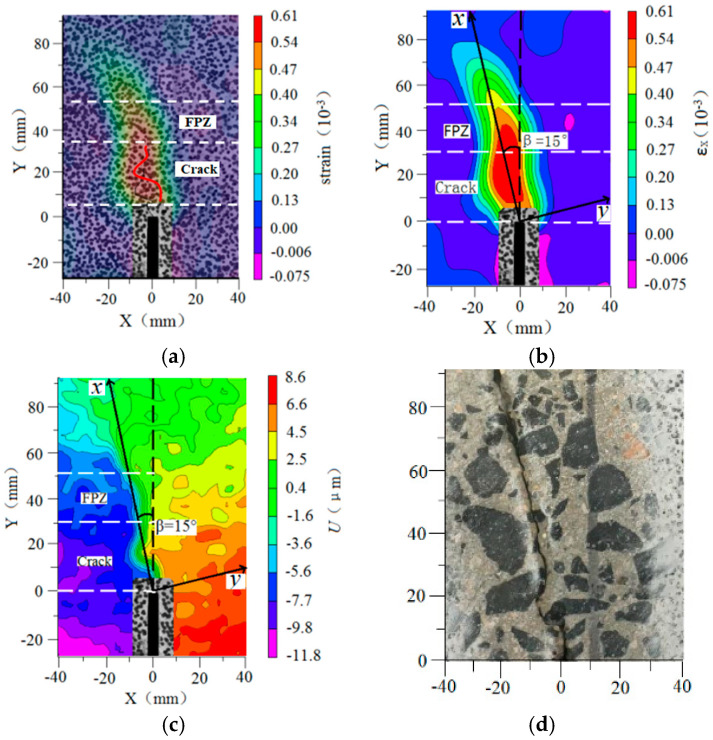
Determination of fracture process zone. (**a**) Post of load peak; (**b**) Strain field; (**c**) Displacement field; (**d**) The direction of crack.

Figure 5 shows the displacement field and strain field of four-point shearing beams using DIC at the post-peak load of 95%. It represents and identifies the characteristics of Mixed-Mode I-II FPZ by the horizontal displacement field. According to the actual image along with the comparison between the horizontal displacement field and the horizontal strain field, the position of the crack tip was located at *Y* = 30 mm, which is the vertical distance from the crack tip to the pre-notch. The core of the strain field is not the FPZ, and it includes the tip of the crack, as shown in Figure 5a,b. The displacement field shown in Figure 5c reveals the parallel distribution of displacement contour lines at the tip of notch. Additionally combining Figure 5a, it presents that the parallel distributed region of displacement field is the crack propagation, and the fan-shaped expanded region of displacement field at the range of the crack tip is the FPZ. The direction of the crack propagation is the connecting line of the contour bump in the local strain field.

**Figure 6:**
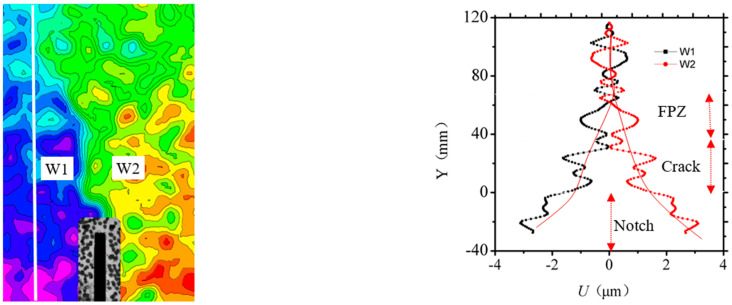
Horizontal displacement developments at different positions above the notch.

In Figure 6, it is shown the developments of the horizontal displacements in the vertical cross-sections on both sides of the notch at the post-peak load of 95%. The horizontal displacements in the two sections of *W*_1_ and *W*_2_ decrease along with the vertical distance to the notch. Additionally, the horizontal displacements present the random fluctuated characteristics by the aggregate distribution in concrete. It can characterize the position and length of Mixed-Mode I-II FPZ in Figure 6, as *L*_FPZ_ = 24.9 mm.

### 3.3. Characteristic of the FPZ

**Figure 7:**
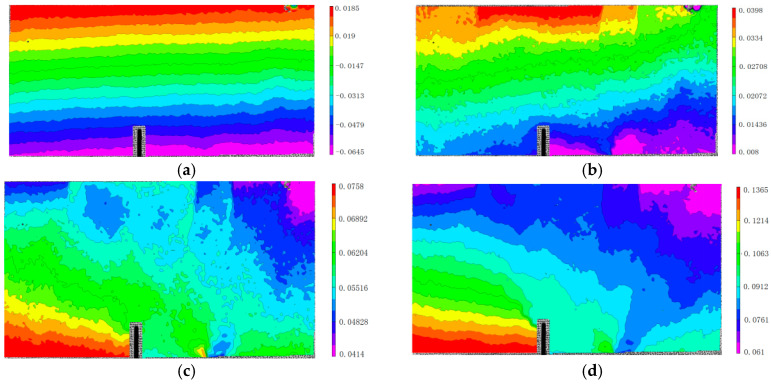
Horizontal displacement fields under different load levels. (**a**) the pre-peak load of 30%; (**b**) the pre-peak load of 50%; (**c**) the pre-peak load of 85%; (**d**) the pre-peak load of 100%.

It presents the evolution of FPZ and explores the development process of the horizontal displacement for a four-point shearing test under Mixed-Mode I-II loading. At the pre-peak load of 30%, the horizontal displacements around the pre-notch mainly show symmetrical distribution, and the variation of the displacement contour is relatively uniform, which is ascribed to elastic deformation, as shown in Figure 7. The horizontal displacement field can determine the neutral axis of the four-point shearing beam. The presence of the pre-notch does not change the variation pattern of the displacement field. The horizontal displacement variation resembles the pure Mode I fracture model. Due to the influence of coarse aggregate distribution, the horizontal displacement field fluctuates locally, as shown in Figure 7a. While, in Figure 7b, at the pre-peak load of 50%, the horizontal displacement filed swerves to the left due to the notch and a larger horizontal displacement gradient appears on the left side of the pre-notch. The horizontal displacement on the left side of the notch is 81 μm, and 64 μm for the right, which is provided by the Mode II component. The distorted displacement contours are also affected by the Mode II component. When it is at the pre-peak load of 85%, the displacement discontinuous zone occurs near the pre-notch, which continues to expand along with the load, as shown in Figure 7c. Figure 7d shows that, at the pre-peak load of 100%, the horizontal displacement contour is offset due to the effect of Mixed-Mode I-II loading and the displacement is greater on the left side of the notch. However, the horizontal displacement converges at Mixed-Mode I-II FPZ of concrete. The displacement of this zone is discontinuous and the inclination angle from the vertical direction is *β* = 75°.

**Figure 8:**
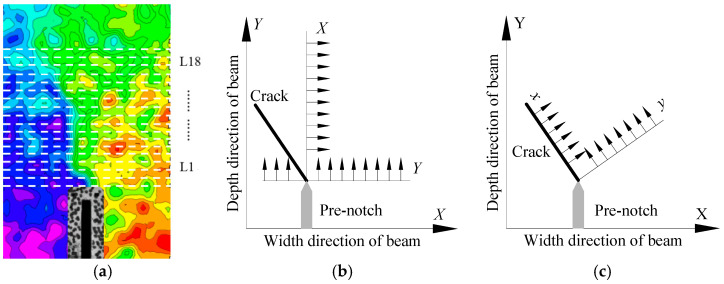
Transformation of the displacement field. (**a**) 18 horizontal sections (**b**) the horizontal and vertical displacement (**c**) the positive and tangential displacement.

It investigates the evolution of FPZ in the four-point shearing beams with pre-notch under the Mixed-Mode I-II loading, and selects a horizontal section of 6 mm from the top of the pre-notch and then increased uniformly by 4 mm, for a total of 18 horizontal sections, denoted by L_1_, L_2_, ..., L_18_, as shown in Figure 8a. This paper mainly presents the Mixed-Mode I-II fracture behavior of the four-point shearing beams, so it is more significant to obtain the positive and tangential displacement at the crack than the horizontal and vertical displacement. Therefore, the global coordinate system (*X*,*Y*) on the experimental plane of the four-point shearing beam and the local coordinate system (*x*,*y*) on the position of crack are determined. The origin of both coordinate systems is at the top of the pre-notch, *X* is the width direction of the beam, *Y* is the depth direction of the beam, *x* is perpendicular to the direction of the crack, and the tangential displacement direction is perpendicular to the positive direction that is shown in Figure 8b,c. Δ*U*_1_ and Δ*V*_1_ are the positive and tangential displacement increments.

**Figure 9:**
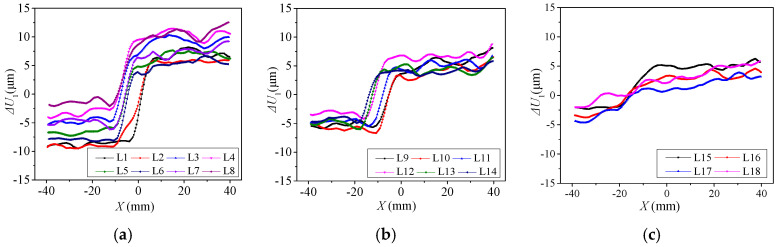
The positive displacements on both sides of the notch. (**a**) Δ*U*_1_ at the horizontal sections of L1 to L8. (**b**) Δ*U*_1_ at the horizontal sections of L9 to L14 (**c**) Δ*U*_1_ at the horizontal sections of L15 to L18.

At the post-peak load of 95%, the Δ*U*_1_ at the top of the pre-notch is about five times greater than that in other regions, as shown in Figure 9. Additionally, the size and distribution of coarse aggregate result in fluctuation of positive displacement. The Δ*U*_1_ at the 58 mm at the top of the pre-notch tip is the same. The crack emerges at 34 mm from the top of the pre-notch tip, as shown in Figure 5a and Figure 9a. The Δ*U*_1_ linearly increases located at the tip of the crack. The linearly increasing area of Δ*U*_1_ coincides with the contour area of positive displacement in Figure 9c. The fluctuation of Δ*U*_1_ in other areas is mainly due to the influence of aggregate distribution. It shows the Δ*U*_1_ in the range of *Y* = 34 mm to *Y* = 58 mm, the increase in Δ*U*_1_ is also linear in Figure 9b. However, the fluctuation is about ±5 μm, which is approximately 0.5 times to the crack. This area is identified as the FPZ of Mixed-Mode I-II crack, with a vertical length of 24 mm. The length of the FPZ is the vertical length divided by the cosine of the angle *β* = 15°. The length of the FPZ of Mixed-Mode I-II crack is *L*_FPZ_ = 24.9 mm.

**Figure 10:**
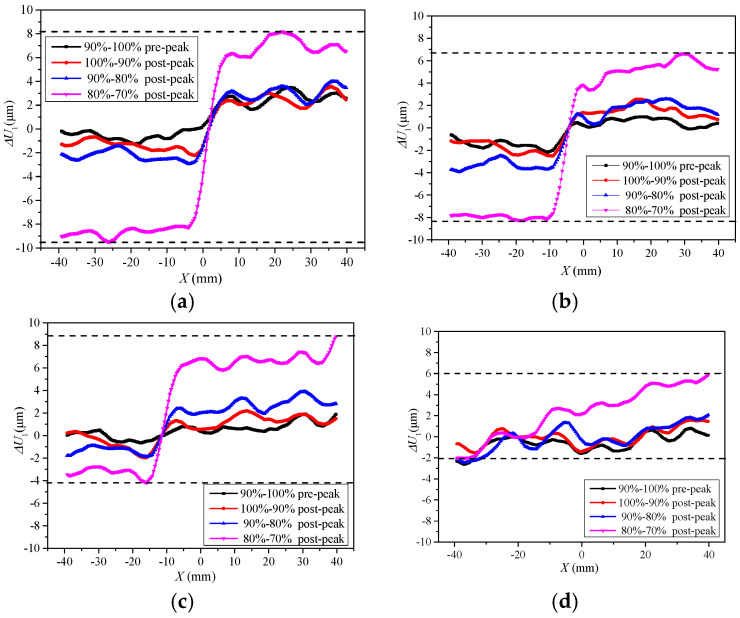
Positive displacements at various load levels. (**a**) *h* = 6 mm; (**b**) *h* = 27 mm; (**c**) *h* = 51 mm; (**d**) *h* = 74 mm.

It shows the L_1_, L_6_, L_12_, and L_18_ sections with distances from the notch tip of 6 mm, 27 mm, 51 mm, and 74 mm in Figure 10a, and analyzes the evolution of positive displacement increments of each section under different load levels. The positive displacement increments of L_1_, L_6_, L_12_, and L_18_ sections change with the variation of load level, as shown in Figure 10. The intersection point of positive displacement increment curves of each section is in the core of the FPZ region, where cracks will penetrate. With the increase in the section of height *h*, the positive displacement increments on both sides of pre-notch decrease gradually at the same loading level, and the intersection point of positive displacement increment curves move to the left, and the FPZ also moves to the left. With the increase in the load level, the difference of positive displacements between the two sides of the pre-notch shows an increasing trend.

**Figure 11:**
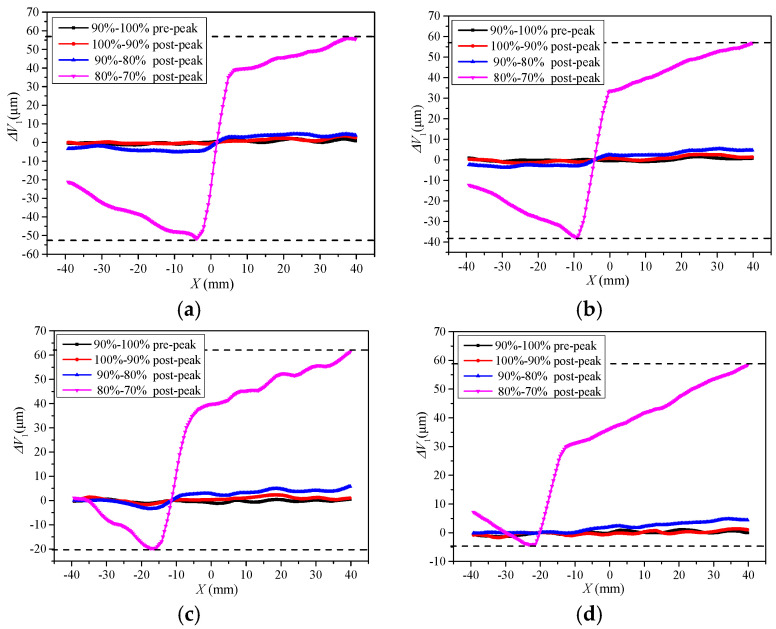
Vertical displacements at various load levels. (**a**) *h* = 6 mm; (**b**) *h* = 27 mm; (**c**) *h* = 51 mm; (**d**) *h* = 74 mm.

Similarly, it analyzes the tangential displacement increments of L_1_, L_6_, L_12,_ and L_18_ sections. The tangential displacement increments of different sections also change with the variation of load level, as shown in Figure 11. Before the post-peak load of 80%, the maximum value of vertical displacement increment at the two sides of the notch is 9.3 μm, and it is 103 μm at the post-peak load of 80% to 70%. In 6 mm to 74 mm from the pre-notch tip, the intersection point of the vertical displacement increment curves changes 0 mm to 20 mm at the horizontal position, and the fracture direction changes about 16°, which is consistent with the direction of the FPZ presented in Figure 5.

The Δ*U*_1_ and Δ*V*_1_ show discontinuity in the FPZ in the pre-peak load of 90% to 100%, as shown in Figure 10 and Figure 11. Additionally, the displacement increment fluctuates around zero. It indicates that the tangential displacements at both sides of the pre-notch are small, and also reveals that the Mode II displacement method is not involved. Therefore, it is shown that the fracture of the four-point shearing beam with the pre-notch is the Mode I at the beginning of the Mixed-Mode I-II loading. The Δ*V*_1_ shows a dislocation offset on both sides of the pre-notch at the post-peak loading of 100~90% to failure. Therefore, it presents that the crack initiation of the four-point shearing beam with the pre-notch is dominated by mode I load, and the effect of the mode II load increases as the loading progresses. Additionally, crack propagation and fracture are caused by the combined effect of Mixed-Mode I-II loading.

### 3.4. The Effect of Coarse Aggregate on the FPZ

**Figure 12:**
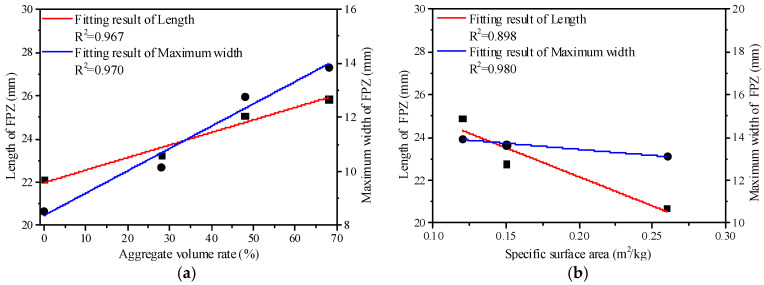
The effect of coarse aggregate on FPZ size. (**a**) FPZ size versus aggregate volume rate; (**b**) FPZ size versus the specific surface area.

Figure 12 shows the length and width of the Mixed-Mode I-II FPZ. The length and maximum width of FPZ present an increase with the volume rate of coarse aggregate, as shown in Figure 12a. The crack propagation has to pass a longer path to bypass the coarse aggregate with the increase in the volume rate of coarse aggregate and needs more energy due to the pulling out of coarse aggregate, the interface friction of the aggregate and mortar, and the cohesive bridging of the coarse aggregate. Accordingly, coarse aggregate can increase ductility in the fracture process. It shows that the size of the Mixed-Mode I-II FPZ conforms to the linear positive correlation with the volume ratio of coarse aggregate [35,36,37,38].

It presents the specific surface area of the aggregate of 0.26 m^2^/kg, 0.15 m^2^/kg, and 0.12 m^2^/kg in mixed proportions of concrete. It shows that the size of the Mixed-Mode I-II FPZ conforms to the linear negative correlation with the specific surface area of coarse aggregate presented in Figure 12b. The fracture rate of coarse aggregate on the plane of fracture is related to the particle size of coarse aggregate, and coarse aggregate with smaller particle size was more likely to fracture. The amount of coarse aggregate with small particle size is increasing in the specific surface area of coarse aggregate, so the amount of broken coarse aggregate on the fracture plane is also increasing. Additionally, the pulling out of coarse aggregate, the interface friction of the aggregate and mortar, and the cohesive bridging of the coarse aggregate are decreasing, and the brittleness of concrete is increasing [39,40].

## 4. Conclusions

In this paper, the FPZ of mixed-mode I-II crack was studied by four-point shearing tests combining with the Lagrange strain monitoring results of DIC technology. Additionally, the influences of volume rate and specific surface area for coarse aggregate on the size of FPZ was discussed at the meso-level. The main results can be summarized, as follows:

1. The process from initiation to failure of concrete mixed-mode I-II fracture can be clearly captured by DIC. The horizontal displacement field can be used to determine the size and shape of the concrete mixed-mode I-II FPZ. The length of the mixed-mode I-II FPZ is the ratio of the height to the cosine of the angle.
2. In the mixed I-II loading, at 30% of the pre-peak stage, the horizontal displacement distribution of the four-point shear beam is consistent with the mode I loading. Beyond 50% of the pre-peak stage, the horizontal displacement contour line is offset due to the influence of the mode II loading and notch. This ultimately converges to an area which is the direction of crack expansion.
3. According to the analysis results for the positive displacement field and the tangential displacement field, under the mixed-mode I-II loading, crack initiation at the pre-notch for four-point shear beams is dominated by the mode I loading, while crack expansion and failure are caused by the combined effect of the mixed-mode I-II loading.
4. The size of the Mixed-Mode I-II FPZ conforms to the linear positive correlation with the volume rate of coarse aggregate, and the linear negative correlation with the specific surface area of coarse aggregate.

## 5. Limitations and Future Recommendation

1. In this paper, the evolution of concrete mesoscale Mixed Mode I-II FPZ in fracture process was studied. The influences of aggregate volume rate and aggregate specific surface area on the size evolution of FPZ were analyzed and discussed. However, when carrying out experimental research, only a single loading method was tested, and the influences of the ratio of Mode I and Mode II on the evolution of FPZ were not carried out, which needs to be studied in the future.
2. In this paper, the research objects on the fracture characteristics of concrete are only focused on plain concrete. Additionally, it is suggested that the influence of reinforcement on the fracture characteristics of structures be considered in future research.

## Figures and Tables

**Table 1:** Chemical constituent of cement (%) [31].

SiO_2_	Al_2_O_3_	Fe_2_O_3_	CaO	MgO	SO_3_	R_2_O	Loss on Ignition
21.18	5.42	3.66	64.28	1.75	2.68	0.91	1.65

**Table 2:** Physical properties of cement [31].

Standard Consistency (%)	SSA (cm^2^/g)	Density (g/cm^3^)	Setting Time (min)		Flexural Strength (MPa)		Compressive Strength (MPa)	
			Initial	Final	3 Days	28 Days	3 Days	28 Days
29	2475	3.17	60	360	4.7	8.9	18.9	48.5

**Table 3:** Quality index of fly ash (%) [31].

Quality Index	Fineness	Loss on Ignition	Water Content	Water Demand Ratio	SO_3_
I-type standard	12	5	1	95	3
The measured results	11	0.38	0.2	89	0.78

**Table 4:** Mix proportions of concrete and compressive strength (kg/m^3^) [31].

MIX	Cement	Fly Ash	Coarse Aggregate			Fine Aggregate	Water	Super-Plasticizer	Volume Rate (%)	SSA (m^2^/kg)	Compressive Strength (MPa)
			4.75–9.5	9.5–19	19–26.5						
S1	715.1	143.0	-	-	-	1286.5	291.7	13.7	0	-	35.5
S2	583.7	116.7	135.0	315.0	-	1050.2	238.2	11.2	28	-	39.2
S3	496.2	99.2	225.0	525.0	-	892.6	202.4	9.5	48	-	47.5
S4	400.0	80.0	323.8	755.6	-	719.6	163.2	7.7	68	-	57.2
S5	400.0	80.0	-	1079.0	-	719.6	163.2	7.7	68	0.12	42.5
S6	400.0	80.0	359.5	479.4	239.8	719.6	163.2	7.7	68	0.15	52.5
S7	400.0	80.0	1079.0	-	-	719.6	163.2	7.7	68	0.26	39.6
